# Abolishing Tau cleavage by caspases at Aspartate^421^ causes memory/synaptic plasticity deficits and pre-pathological Tau alterations

**DOI:** 10.1038/tp.2017.165

**Published:** 2017-08-08

**Authors:** F Biundo, C d'Abramo, M D Tambini, H Zhang, D Del Prete, F Vitale, L Giliberto, O Arancio, L D'Adamio

**Affiliations:** 1 Department of Microbiology and Immunology, Albert Einstein College of Medicine, Bronx, NY, USA; 2 Litwin-Zucker Center for Research in Alzheimer's Disease, Feinstein Institute for Medical Research, Northwell Health, Manhasset, NY, USA; 3 Department of Pathology and Cell Biology and Taub Institute for Research on Alzheimer's Disease and the Aging Brain, Columbia University, New York, NY, USA

## Abstract

*TAU* mutations are genetically linked to fronto-temporal dementia (FTD) and hyper-phosphorylated aggregates of Tau form neurofibrillary tangles (NFTs) that constitute a pathological hallmark of Alzheimer disease (AD) and FTD. These observations indicate that Tau has a pivotal role in the pathogenesis of neurodegenerative disorders. Tau is cleaved by caspases at Aspartate^421^, to form a Tau metabolite known as δTau; δTau is increased in AD, due to the hyper-activation of caspases in AD brains. δTau is considered a critical toxic moiety underlying neurodegeneration, which initiates and facilitates NFT formation. As Tau is a therapeutic target in neurodegeneration, it is important to rigorously determine whether δTau is a toxic Tau species that should be pharmacologically attacked. To directly address these questions, we have generated a knock-in (KI) mouse called *Tau*^*DN*^—that expresses a Tau mutant that cannot be cleaved by caspases. *Tau*^*DN*^ mice present short-term memory deficits and synaptic plasticity defects. Moreover, mice carrying two mutant *Tau* alleles show increased total insoluble hyper-phosphorylated Tau in the forebrain. These data are in contrast with the concept that δTau is a critical toxic moiety underlying neurodegeneration, and suggest that cleavage of Tau by caspases represents a negative feedback mechanism aimed to eliminate toxic Tau species. Alternatively, it is possible that either a reduction or an increase in δTau leads to synaptic dysfunction, memory impairments and Tau pathology. Both possibilities will have to be considered when targeting caspase cleavage of Tau in AD therapy.

## Introduction

The microtubule-associated protein Tau (MAPT) is a soluble protein that promotes assembly and stabilization of microtubules. In neurons, Tau is required for vesicular transport and axonal growth. Recent data suggest that a fraction of Tau protein is localized extracellularly.^1, 2, 3, 4^ However, the biological function of extracellular tau is unknown.

The involvement of Tau in neurodegenerative disorders is clear: hyper-phosphorylated aggregates of Tau are the main components of neurofibrillary tangles (NFTs), which, together with amyloid plaques and neuronal and synaptic loss, constitute the primary pathological hallmarks of Alzheimer disease (AD). Furthermore, several *TAU* mutations are genetically linked to fronto-temporal dementia (FTD).^5, 6, 7, 8, 9, 10, 11, 12^

Tau expression is regulated by alternative splicing in a species-specific, developmental- and tissue-dependent manner.^13, 14, 15, 16^ Depending upon the inclusion or exclusion of exon 10, Tau isoforms with three or four microtubule binding domains are generated (3R and 4R, respectively). In the adult human brain, splicing is balanced with a 1:1 expression of the 3R and 4R Tau isoforms. In contrast, mouse brains express only 3R Tau at birth and only 4R Tau at adulthood. Because of these differences, it is argued that human Tau, but not murine Tau, can exert neurotoxic effects. However, this hypothesis is contrasted by data showing that endogenous mouse Tau is required for Aβ-induced postsynaptic dysfunction and behavioral defects,^17, 18, 19, 20, 21, 22, 23, 24^ which suggest that murine Tau can carry out pathogenic functions that resemble that of human Tau AD.

Caspases are activated in AD,^25^ and can process proteins involved in AD including amyloid precursor protein,^26, 27, 28^ Presenilins^29, 30^ and Tau.^20^ Tau is cleaved at Aspartate^421^ (D^421^) by caspases into two peptides. Although the short COOH-terminal Tau peptide has not been the subject of investigation, the NH2-terminal Tau fragment, called δTau, has been extensively analyzed. It is widely believed that δTau facilitates Tau aggregation into insoluble forms and the formation of NFTs.^20, 21, 31, 32, 33^ Hence, caspase-mediated Tau cleavage is viewed as an early pathological event triggering NFT pathology and δTau as a critical toxic moiety underlying neurodegeneration.^21, 31, 34, 35^ However, the data supporting a pathogenic role of δTau are correlative and/or based on aberrant overexpression of Tau and δTau.^34, 36, 37^ Thus, the possibility that cleavage of Tau by caspases represents a negative feedback mechanism aimed to eliminate toxic forms of Tau and/or to generate ‘beneficial’ Tau fragments must still be considered.

On the basis of the premise that murine Tau can reproduce the toxicity of human Tau, we have tested in mice whether δTau is a toxic Tau species. To this end, we have generated knock-in (KI) mice in which the endogenous Tau codon GAC in exon 12, encoding for D^421^ has been mutated into AAC, which now encodes for an Asparagine (N). These KI mice, called *Tau*^*DN*^, express a Tau mutant that cannot be cleaved by caspases at position D^421^ and therefore cannot generate δTau. Analysis of biochemical Tau modifications, learning, memory and synaptic plasticity in these KI mice should help elucidate the functional consequences of Tau cleavage by caspases. If this cleavage promotes Tau hyper-phosphorylation and insolubility, insoluble forms of hyper-phosphorylated Tau should be reduced in *Tau*^*DN*^ mice as compared with the control mice. Moreover, if the Tau fragments produced by caspases are neurotoxic and damage synaptic plasticity and memory, *Tau*^*DN*^ mice should not present synaptic/memory deficits. In contrast, if cleavage of Tau by caspases eliminates harmful forms of Tau and/or produces Tau fragments that are functionally beneficial, *Tau*^*DN*^ mice should accumulate more insoluble forms of hyper-phosphorylated Tau and may develop deficits in synaptic plasticity, learning and memory.

## Materials and methods

### Mice and ethics statement

Mice were handled following the Ethical Guidelines for Treatment of Laboratory Animals of Albert Einstein College of Medicine (AECOM). The procedures were described and approved by the Institutional Animal Care and Use Committee at the AECOM in animal protocol number 20130509.

### Generation of *Tau*^*DN*^ KI mice

#### Vector design outline

A ~8.49 kb region used to construct the targeting vector was sub-cloned from a positively identified C57BL/6 BAC clone (RP23: 83F10). The region was designed such that the long homology arm (LA) extends ~6.11 kb 5′ to the site of the point mutations (G>A) in exon 12 and the FRT-LoxP-flanked Neo cassette is inserted 462 bp 3′ to the point mutation. The short homology arm (SA) extends 1.91 kb 3′ to the FRT-LoxP-flanked Neo cassette. The targeting vector was constructed using Red/ET recombineering technology. The BAC was sub-cloned into a ~2.4 kb backbone vector (pSP72, Promega, Madison, WI, USA) containing an ampicillin selection cassette for re-transformation of the construct prior to electroporation. A pGK-gb2 FRT-LoxP Neo cassette was inserted into the gene as described in the project schematic (Supplementary Figure 1a). The targeting construct can be linearized using *Not*I prior to electroporation into embryonic stem (ES) cells. The total size of the targeting construct (including vector backbone and Neo cassette) is 14.21 kb. The schematic representation of the targeting vector is shown in Supplementary Figure 1a.

#### Generation of the point mutation

The mutation was engineered by overlap extension PCR. Two primary PCR fragments that overlap 16 bp 5′ of the G>A point mutation were generated using primers PT5/PT2 and PT3/PT4. The schematic is shown in Supplementary Figure 1b. Oligos used to generate the mutations are shown below:

PT5: 5′-GCAAAGCCCTGTGTACATTGTTCC-3′

PT2: 5′-CACCATGTCGATGCTGCCCGTGGAAG-3′

PT3: 5′-CAGCATCGACATGGTG**A**ACTCACCACAGCTTGCCAC-3′

PT4: 5′-CCTAAAGTCCCAGGTCTGTAATGGTG-3′

The G>A point mutation was engineered into primer PT3 (the mutation is indicated in bold and underlined). The two primary products were then mixed and used as a template in a secondary PCR in which PT5/PT4 primer pair amplifies the entire sequence containing the point mutation. The endogenous 5′ *Eco*RI and endogenous 3′ *Kpn*I restriction sites were used for insertion of the mutant PCR fragment into the sub-cloned construct. The targeting vector was confirmed by restriction analysis after each modification step and by sequencing using primers designed to read from the selection cassette into the SA (N1) and the genomic sequence containing the point mutations (N2). P6 and T73 primers anneal to the BAC sub-clone sequence and read into the 5′ and 3′ ends of the sub-cloned vector. Also, sequencing results with primers SQ1 confirmed that no error was introduced into the PCR-modified region. Primers used for sequencing are shown below:

Primer P6: 5′-GAGTGCACCATATGGACATATTGTC-3′

Primer T73: 5′-TAATGCAGGTTAACCTGGCTTATCG-3′

Primer N1: 5′-TGCGAGGCCAGAGGCCACTTGTGTAG-3′

Primer N2: 5′-TTCCTCGTGCTTTACGGTATCG-3′

Primer SQ1: 5′-ACCACAGACTAGCTCTGGTGTATAG-3′

#### Generation, screening and confirmation of recombinant ES clones

Ten micrograms of the targeting vector was linearized by *Not*I and then transfected by electroporation of C57Bl/6 (B6) ES cells. After selection with G418 antibiotic, surviving clones were expanded for PCR analysis to identify recombinant ES clones. Screening primer A2 was designed downstream of the SA outside the 3′ region used to generate the targeting construct. PCR using A2 with the F3 primer (located within the Neo cassette) amplifies a 2.29 kb fragment. Clones 184, 281, 284, 331 and 351 were identified as positive, expanded and reconfirmed for SA integration. Confirmation of the point mutation was performed by PCR using the SQ1 and N2 primers. This reaction produces a 0.94 kb-product. Sequencing was performed on purified PCR DNA to confirm presence of the point mutation using the SQ1 primer. Schematic of the strategy and PCR results are shown in Supplementary Figures 2a and c. Primers for PCR screening are shown below:

SQ1: 5′-ACCACAGACTAGCTCTGGTGTATAG-3′

F3: 5′-GCATAAGCTTGGATCCGTTCTTCGGAC-3′

A2: 5′-GTTCTTTAATGGGAAAGGCTGGCTG-3′

N2: 5′-TTCCTCGTGCTTTACGGTATCG-3′

Sequence from a confirmed clone is shown below. The point mutation is in bold and underlined.

TCACCCGTGGTGTCTGGGGACACATCTCCACGGCACCTCAGCAATGTGTCTTCCACGGGCAGCATCGACATGGTG**A**ACTCACCACAGCTTGCCACACTAGCCGATGAAGTGTCTGCTTCCTTGGCCAAGCAGGGTTTGTGATCAGGCTCCCAGGGCAGTCAATAATCATGG

**Figure 1:**
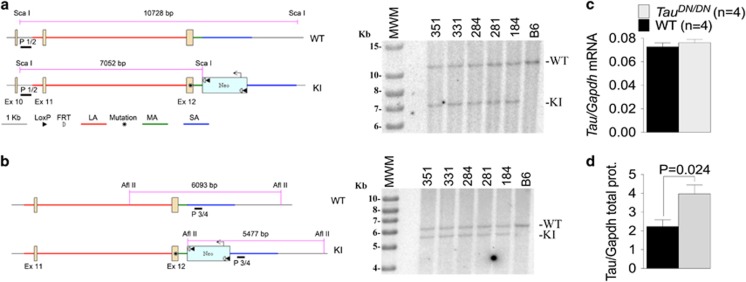
Generation and characterization of *Tau*^*DN*^ mice. Confirmation of positive embryonic stem (ES) clones by Southern blotting analysis. (**a**) Long homology arm (LA) analysis. Southern blot analysis of *Sca*I-digested genomic DNA with P1/2. Schematic is represented on the left and the Southern blot is shown on the right. The wild-type (WT) band is 10 728 bp, whereas the knock-in (KI) band is 7052  bp. (**b**) Short homology arm (SA) analysis. Southern blot analysis of *Afl*II-digested genomic DNA with P3/4. Schematic is represented on the left and the Southern blot is shown on the right. The WT band is 6093 bp, whereas the KI band is 5477 bp. (**c**) Quantification of total brain *Tau* mRNA levels shows similar level of *Tau* expression is in WT and *Tau*^*DN/DN*^ littermates. (**d**) Quantification of total Tau protein shows that *Tau*^*DN/DN*^ mice present higher levels of total Tau as compared with the WT littermates. The samples analyzed in **c** and **d** are derived from the same animals (half brain was used for RNA extraction, the other half for protein preparation). The animals were littermates and were analyzed at 3 weeks of age.

Clones 184, 281, 284, 331 and 351 were further analyzed by Southern blot. Genomic DNA was digested with either *Sca*I or *Afl*II, separated on a 0.8% agarose gel and transferred to a nylon membrane. DNA from C57Bl/6 (B6), mouse strain was used as a wild-type control. The *Sca*I-digested DNAs were hybridized with a probe targeted against the 5′ external region (P1/2, schematic and expected sizes are indicated in Figure 1a) and the *Afl*II-digested DNAs were hybridized with a probe targeted against the 3′ internal region (P3/4, schematic and expected sizes are indicated in Figure 1b). Primers and probe sequences are shown below:

External LA primers:

P1 5′-GTCTGTCTCTGTTGTGAGCCTAGAC-3′

P2 5′-AGGAACATTCTGTAAGCCACAGCC-3′

P1/2 Probe sequence (413 bp)

GTCTGTCTCTGTTGTGAGCCTAGACAAGGGGTGGAGGAAATGGTTTTATATCCCATCCTGTGGCTCTCCAGAAAAGTCTGTATAAAAGGACCAGCCATGGGTTTGATTGGGTCCCTCCCACCCCCAAAGGCCGGCTCTGTGAACATCTCAGCTTTGTCACAGCCAACATGGGGGACACTTCAATTGCATCAGCTATAACATGAGAACTTTTGGGCTGTTGTCTCATCTTCGCTGTAACATTGTTCAAAGAAAGCATTCTTCCCAGGAAAACAGGCCAGGTCACTCCCTCTCCTGCTATACCCTAGTGTGGTGACCTGAGCCACAGGGGAGGTTAGGGTGTCTCTCCTCATGGTGCATTTGACAGGCGGTTTGATTCCAAGGTCTTGAGGGGCTGTGGCTTACAGAATGTTCCT

Internal SA primers:

P3 5′-GCT CAG ATG CCT GCT TGA TAA AGC-3′

P4 5′-CCT AAA GTC CCA GGT CTG TAA TGG TG-3′

P3/4 probe sequence (311 bp) GCTCAGATGCCTGCTTGATAAAGCACTGTGGGGGTAACGTGGGTGTGTGTGCCCCTTCTGCAGGGCAGCCTGTGGGAGAAGGGGTATTGGGCAGAAGGAAGGTAAGCCAGCAGGTGGTACCTTGTAGATTGGTTCTCTTGAAGGCTGCTCTTGACATCCCAGGGCACTGGCTTCTTCCTCCCTCCCCGCAAGGTGGGAGGTCCTGAGCGAGGTGTTTCCCTTCGCTCCCACAGGAAAAGCTGCTTTACTGAGTTCTCAAGTTTGGAACTACAGCCATGATTTGGCCACCATTACAGACCTGGGACTTTAGG

#### Tau^DN^ KI mice

Targeted iTL IC1 (C57BL/6) embryonic stem cells were microinjected into Balb/c blastocysts. Resulting chimeras with a high percentage of black coat color were mated to C57BL/6 FLP mice to remove the Neo cassette. Tail DNA was analyzed from pups with black coat color for: (1) deletion on the Neo cassette; (2) presence of the point mutation; (3) presence of the FLP transgene; (4) confirmation of the SA integration. Schematic of the deleted allele is shown in Supplementary Figure 3a, primers used for these screenings are shown below:

NDEL1: 5′-GGCTGACCTGCTTGTCACCTAAC-3′

NDEL2: 5′-CCTAAAGTCCCAGGTCTGTAATGGTG-3′

FLP1: 5′-CACTGATATTGTAAGTAGTTTGC-3′

FLP2: 5′-CTAGTGCGAAGTAGTGATCAGG-3'

SQ1: 5′-ACCACAGACTAGCTCTGGTGTATAG-3′

F7: 5′-GGAACTTCGCTAGACTAGTACGCGTG-3′

F3: 5′-GCATAAGCTTGGATCCGTTCTTCGGAC-3′

A2: 5′-GTTCTTTAATGGGAAAGGCTGGCTG-3′

The PCR showing deletion of the Neo cassette is shown in Supplementary Figure 3b. Sequence of the deleted PCR product representative mouse (#155) shows (see below) the deletion of the Neo cassette with the exception of one set of LoxP-FRT sites and the remaining section of the Neo. The FRT site is underlined, the loxP site is bolded and the Neo fragment is shaded. TTCAGTCCCCACTCACACCCACACAAGTTAACAGCACCTGCCTACGGCCCCACGAACACACCAAGTCTCAAATCTCTCATTGCTGCCACTGTCCCTGAAGCCCCTAGGATGGGGCTATGGGCAATTAGCTGCCCTACGTACGGTGTTGACGAGGCGTCCGAAGAACGGATCCAAGCTTATGCATGAATTCTGCAGGTACC**ATAACTTCGTATAATGTATGCTATACGAAGTTAT**GTTCGAACGAAGTTCCTATTCTCTAGAAAGTATAGGAACTTCGCTAGACTAGTACGCGTGTACACTTAAGCCGGCGTACGTGCACAGTACTGTCCCTCAGCCACTCCCCAGAAGCAGCCTCCAGAGCCTTCTTCACCCTCTAATACTCAGAGAGGGAGGGCGGGGTCAGGGGGGGGAA

The PCR product amplified by primers SQ1 and F7 (Supplementary Figure 3c) was sequenced to verify the introduction of the point mutations. SQ1 is located on the LA, 5′ of the point mutation. F7 is located inside the Neo cassette. The amplified size for SQ1/F7 is 732 bp. Sequencing was performed on purified PCR DNA to confirm presence of the point mutation using the SQ1 primer. Sequencing from mouse 155 is shown below. The point mutation G→A is bolded and underlined. ACCATGGAGCAGAAATTGTGTATAAGTCACCCGTGGTGTCTGGGGACACATCTCCACGGCACCTCAGCAATGTGTCTTCCACGGGCAGCATCGACATGGTG**A**ACTCACCACAGCTTGCCACACTAGCCGATGAAGTGTCTGCTTCCTTGGCCAAGCAGGGTTTGTGATCAGGCTCCCAGGGCAGTCAATAATCATGGAGAGAAGAGAGAGTGAGAGTGTGG

Primer set FLP1 and FLP2 was used to screen mice for presence of the FLP transgene. The amplified product for primer set FLP1 and FLP2 is 725 bp (Supplementary Figure 3d). Tail DNA samples from correctly targeted mice were amplified with primers F3 and A2. F3 is located inside the Neo cassette and A2 is located downstream of the SA, outside the region used to create the targeting construct. F3/A2 amplifies a fragment of 2.28 kb in length (Supplementary Figure 3e).

### Analysis of *Tau* mRNA levels

Mouse brains were removed and divided at the midline so that one half of brain was processed for RNA extraction (the other half has been used for protein preparation, see below). Total RNA was prepared using RNeasy Mini Kit (Qiagen, Hilden, Germany, 74104) and RNA integrity was confirmed using an Agilent Bioanalyzer (Santa Clara, CA, USA). cDNA was synthesized using High-Capacity cDNA Reverse Transcription Kit (Invitrogen, Carlsbad, CA, USA, 4368814). Real-time PCR was performed with ABI TaqMan probes to murine *Tau* (Mm00521988_m1) and *Gapdh* (Mm00521988_m1), using 7900HT Fast Real-Time PCR System (Applied Biosystems). RT-PCR data was analyzed by LinRegPCR software (Amsterdam, Nederland). Student’s *t*-test was used for all analyses, with data presented as average (*Tau/Gapdh*)±s.d.

### Western blot analysis for total-Tau

The other half brain tissue was homogenized in 4 ml homogenization buffer (0.25 m sucrose, 20 mm HEPES, 1 mm EGTA, 1 mm EDTA, pH 7.4) plus Phosphatase/Protease inhibitors (Pierce, Waltham, MA, USA) using a glass-teflon tissue grinder. Total homogenate (S1) was centrifuged at 800 *g*, for 15′ at 4 °C. Supernatant (S2) was collected and total protein was quantified by Bradford analysis. Overall, 10 μg of S2 was loaded onto a 4–12% Bis-Tris denaturing gel (Biorad, Hercules, CA, USA, 3450125), and separated by PAGE. Blots were probed for total Tau using DA9 (1:1000, O/N at 4 °C) and for Gapdh using anti-GAPDH (Origene, Rockville, MD, USA, TA308884, 1:10 000, O/N at 4 °C). Secondary antibodies used were HRP-conjugated Goat-anti-mouse (Southern Biotech, Knoxfield, VIC, Australia, OB103105) and Goat-anti-rabbit (Southern Biotech, OB405005), respectively. Blots were developed with West Dura ECL reagent (Thermo Fisher Scientific, Waltham, MA, USA, PI34076) and signals were revealed using ChemiDoc MP Imaging System (Biorad). Signal intensity was quantified with Image Lab software (Biorad), and each Tau lane was normalized to Gapdh. Student’s *t*-test was used for all analyses, with data presented as average (Tau/Gapdh)±s.d.

### *In vitro* caspase cleavage of Tau

Seven micrograms of S2 fraction was added to 1 × Caspase buffer (50 mm HEPES, pH 7.2, 50 mm NaCl, 10 mm EDTA, 5% glycerol, 10 mm DTT), for a final volume of 60 μl. Five units of Caspase-3 (Enzo Life Sciences, Billerica, MA, USA, ALX-201-059-U025) were added and reaction was incubated at 37 °C for 1 h. Samples were denatured in SDS-PAGE loading buffer and separated by PAGE. Blots were probed for cleaved tau using anti-cleaved-Tau-Asp^421^ clone C3 (EMD Millipore, Billerica, MA, USA, 36-017, 1:2000, O/N at 4 °C).

### ELISA for δTau

Ninety-Six well plates (Nunc, Waltham, MA, USA) were coated with anti-Tau caspase cleaved antibody (C3, Millipore) at a final concentration of 6 μg ml^−1^, for 48 h at 4 °C. After washing 3 ×, the plates were blocked for 1 h at room temperature using StartingBlock Blocking buffer (Thermo Scientific). Plates were washed 5 × and 50 μl (1 μg μl^−1^) of brain S2 fraction were added to the wells with 50 μl of DA9-HRP detection antibody. Plates were incubated O/N shaking at 4 °C and then washed 9 × in wash buffer. 1-Step ULTRA TMB-ELISA (Thermo Scientific) was added for 30′ at room temperature before stopping the reaction with 100 μl 2 m H_2_SO_4_. Plates were read with an Infinite m200 plate reader (Tecan, Morrisville, NC, USA) at 450 nm. Recombinant human Tau-441 was purchased from rPeptide (Watkinsville, GA, USA).

### Behavioral experimental procedures

The sample size was pre-determined on the basis of our unpublished data and a recent report testing mice expressing a pathogenic Tau-mutant protein.^38^

Mice were pre-handled by the investigator for 10 days during the 2 weeks preceding the behavioral tests. The behavioral experiments were previously described.^39, 40^ In the elevated zero maze (apparatus from Stoelting, Wood Dale, IL, USA), Y-maze, open field, novel object recognition (apparatus from Stoelting) and Morris water maze, the behavior of mice was monitored using a video camera, and their movements were analyzed with a video tracking system (ANY-maze, Stoelting). Behavioral testing was conducted during the light cycle. On each testing day, animals were transported to a behavioral testing suite in their home cages and allowed to acclimate for at least 30 min prior to the start of testing.

We used pre-established inclusion/exclusion criteria. Animals matching these criteria would be excluded from the analysis. Elevated zero maze: animals that fell off the annular apparatus during the elevated zero maze test. Y-maze: mice that enter less than three arms during the testing stage. Open field: mice that exhibit deficits in ambulation. Novel object recognition: mice that do not explore the objects for more than 20 s either during the training and/or testing stage. Morris water maze: mice that swim close to the wall of the pool (thigmotaxis). However, no animal was excluded from the analysis since no animal met these criteria.

Randomization was used to control unwanted effects that can be introduced through experimental variation that is part of the experimental design but is not being measured (that is, potential different behavior in the morning and afternoon, or preference for left and right side of the apparatus). We used the following methods of randomization. Y-maze: the relative position of the novel vs known arms (that is, left or right) was counterbalanced within each genotype to reduce place preference effects. Novel object recognition: during the familiarization each genotype was alternatively exposed to two sets of familiar object. During the testing position of familiar and novel object was counterbalanced to prevent an eventual preference of each mouse for the left or the right side. Morris water maze: animals were given three daily trials using a random or semi-random set of start locations.

The experimenter was not blind to the genotypes because all measurements were taken automatically by video tracking software.

### Statistical analysis

Statistical analysis was carried out using the Prism software (GraphPad, La Jolla, CA, USA) and was performed by analysis of variance (ANOVA), with one between-subjects factor (genotype) and, when appropriate, a within-subjects factor (for example, day). When significant effects were found, the data were analyzed by *post hoc* comparison tests (Tukey’s or Fisher’s LSD). The level of significance was set at *P*<0.05. Data met assumptions of the tests according to the Shapiro–Wilks test. Variation within each group was estimated as standard error of the mean. Variance is similar between the groups according to Bartlett’s test (ANOVA), F-test (*t*-test).

### Electrophysiological studies

Hippocampal slices were prepared as previously described.^41^ Following assessment of basal synaptic transmission by plotting the stimulus voltages against slopes of field-excitatory post synaptic potentials (fEPSP), baseline was recorded every minute at an intensity that evoked a response 35% of the maximum evoked response. Long-term potentiation (LTP) was induced using a theta-burst stimulation (4 pulses at 100 Hz, with the bursts repeated at 5 Hz and each tetanus including 3 ten-burst trains separated by 15 s). Responses were measured as fEPSP slopes expressed as percentage of baseline.

### Immunohistochemistry

After decapitation, half brain was fixed overnight in 4% paraformaldehyde at 4 °C. Serial sections were cut from the fixed brain with a vibratome, conserved in Tris-buffered saline (TBS) (50 mm Tris, 150 mm NaCl, pH 7.6)/0.02%NaN_3_, and stained on multiwell plates. Endogenous peroxidases were quenched with 3% H_2_O_2_/0.25% Triton X-100/TBS for 30′. Non-specific binding was blocked with 5% Milk-TBS for 1 h at room temperature. Primary antibody PHF1 (1/5000) diluted in 5% Milk-TBS was left overnight at 4 °C, shaking. After 5 × 5′ washes in TBS, samples were incubated with biotin-conjugated secondary antibodies directed against the specific IgG1 isotype diluted 1/1000 in 20% Superblock, for 2 h at room temperature. After 5 × 5 washes in TBS, samples were incubated with Streptavidin-HRP for 1 h. Staining was visualized with 3,3′-diaminobenzidine (Sigma, St. Louis, MO, USA, D5637-5G).

### Brain sample preparation of soluble and insoluble Tau

To measure soluble and insoluble Tau, brains were removed and processed as described.^42, 43^ Briefly, the brain was removed and divided at the midline so that just one half of brain was dissected for biochemical analysis. Forebrain and hindbrain were homogenized separately using an appropriate volume of homogenizing buffer, a solution of TBS, pH 7.4, containing 10 mm sodium fluoride, 1 mm sodium vanadate and 2 mm EGTA, plus a complete Mini protease inhibitor cocktail (Roche, Branford, CT, USA). Brain samples were stored at −80 °C and used for separate measurement of soluble (heat stable preparation=hsp) and insoluble tau. Hsp were made by adding 5% 5m NaCl and 4% β-mercaptoethanol. Samples were heated at 100 °C for 10 min, cooled at 4 °C for 15 min and spun at 14 000 *g* for 10 min. To obtain the insoluble Tau preparation,^44^ 500 μl of homogenate were spun at 6000 *g* for 10′ at 4 °C. The collected supernatant was centrifuged at 200 000 *g* for 30′ at 25 °C. Pellet was resuspended in 450 μl of homogenizing buffer and spun again at 200 000 *g* for 30′ at 25 °C. Final pellet was resuspended in 200 μl of 1 × sample buffer. Insoluble tau preparations were loaded onto Criterion 4–20% Tris-HCl gel (Biorad 3450032), and separated by PAGE. Blots were probed using DA9 antibody (1:1000, O/N at 4 °C). Goat anti-mouse IgG1-HRP (Southern Biotech 1070-05) was used as secondary antibody (1:1000, 1 h RT). Signal was revealed using Immobilion Western Chemiluminescent HRP Substrate (EMD Millipore WBKLS0100).

### Low -Tau sandwich ELISA

Low-Tau sandwich ELISAs were performed as published.^42^ Ninety-six well plates (Nunc) were coated either with DA31 (total Tau), CP13 (pSer^202^), PHF1 (pSer^396-404^) or RZ3 (pThr^231^) at a final concentration of 6 μg ml^−1^, for 48 h at 4 °C. After washing 3 ×, the plates were blocked for 1 h at room temperature using StartingBlock Blocking buffer (Thermo Scientific). Plates were washed 5 × and 50 μl of samples (heat stable and insoluble preparations) were added to the wells, with 50 μl of DA9-HRP detection antibody. Plates were incubated O/N shaking at 4 °C and then washed 9 × in wash buffer. 1-Step ULTRA TMB-ELISA (Thermo Scientific) was added for 30′ at room temperature before stopping the reaction with 100 μl 2m H_2_SO_4_. Plates were read with an Infinite m200 plate reader (Tecan) at 450 nm.

### Antibodies used

DA9, DA31, CP13, RZ3, DA9-HRP and PHF1 have been produced in Dr. Peter Davies’ lab, AECOM and Feinstein Institute For Medical Research, and have been widely used, published and validated; anti-GAPDH (Origene TA308884), Anti-cleaved-Tau-Asp^421^ clone C3 (EMD Millipore 36-017); Goat Anti-Mouse IgG1 Human ads-BIOT (Southern Biotech 1070-08); Streptavidin-HRP; Goat anti-mouse IgG1-HRP (Southern Biotech 1070-05); Goat-anti-mouse (Southern bio, OB103105) and Goat-anti-rabbit (Southern bio, OB405005).

## Results

### Generation of *Tau*^*DN*^ mice

To test whether δTau is a critical toxic moiety underlying neurodegeneration, we have generated *Tau*^*DN*^ KI mice. These mice carry a *Tau* point mutation that changes the codon for D^421^ into one encoding for an N. This mutation is predicted to abolish cleavage by caspases at this site and the production of δTau. To generate the mice, we constructed a targeting vector bearing a mutated C57BL/6 *Tau* exon 12 (Supplementary Figure 1a). The targeting vector was transfected in C57Bl/6 (B6) ES cells. Correct targeting in C57BL/6 ES cell clones was verified by PCR genotyping and sequencing as shown in Supplementary Figure 2 and explained in detail in the ‘Materials and methods’ section. Reconfirmation of correct targeting was performed by Southern blot analysis. As shown in Figures 1a and b, Southern blot analysis of *Sca*I-digested genomic DNA from targeted ES clones with a probe external to the LA (P1/2) yielded the correct WT (10 728 bp) and KI (7052 bp) bands. Likewise, Southern blot analysis of *Afl*II-digested genomic DNA with a probe internal to the SA (P3/4) also yielded the correct WT (6093 bp) and KI (5477) bands.

Targeted C57BL/6 ES cells were microinjected into Balb/c blastocysts. Chimeras with a high percentage black coat color were mated to C57BL/6 FLP mice to remove the Neo cassette. Mice #155 (male), 156 (male), 157 (male), 158 (male) and 160 (female) have been identified for targeted integration, somatic Neo deletion and confirmed with the introduced point mutation (Supplementary Figures 3a, b, c and e, see ‘Materials and methods’ section for sequence results). Mice #156, 158 and 160 are FLP absent (Supplementary Figure 3d). We have produced homozygous-mutant mice (designed as *Tau*^*DN/DN*^) by mating mice #156 and 160. Mating two different founders to generate homozygous mice controls for phenotypic differences due to unwanted and unknown genetic alterations distinct from the targeted *Tau* mutation.

To verify that gene editing did not alter *Tau* gene expression, we isolated RNA from the brain of 3 weeks old *Tau*^*DN/DN*^ and WT littermates. *Tau* mRNA expression was tested by real-time quantitative RT-PCR. As shown in Figure 1c, expression of *Tau* was comparable between the two genotypes, indicating that our gene editing strategy did not alter transcription of the *Tau* gene.

Next, we asked whether the point mutation alters Tau protein levels. We prepared protein homogenates from the same eight animals (four *Tau*^*DN/DN*^ and four WT) used for *Tau* mRNA expression studies. Albeit *Tau* expression was similar in mice of the two genotypes (Figure 1c), total Tau protein levels were significantly higher in *Tau*^*DN/DN*^ mice as compared with WT littermates (Figure 1d). How to reconcile these apparently contradictory results? Soluble Tau^DN^ may be more stable than Tau. Also, Tau^DN^ may be more prone to aggregate into insoluble and long-lived complexes. Finally, mutant *Tau* mRNA may be more efficiently translated as compared with WT *Tau* mRNA. These possibilities are not mutually exclusive.

**Figure 2:**
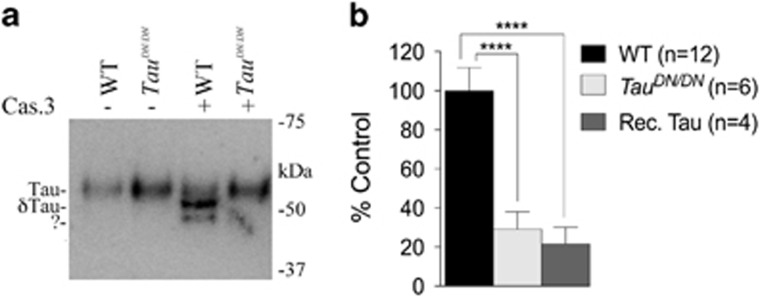
*Tau*^*DN/DN*^ mice do not produce δTau. (**a**) Brain homogenates from 3 week-old *Tau*^*DN/DN*^ and wild-type (WT) littermate mice were treated with (+) or without (−) Caspase-3 prior to western blot analysis with the anti-δTau antibody C3. WT, but not *Tau*^*DN/DN*^, brain lysates show presence of δTau after Caspase-3 digestion. The (?) indicates a band produced by Caspase-3 in WT sample and reactive with C3 of uncertain nature. (**b**) ELISA with C3 shows that δTau is present in WT but not *Tau*^*DN/DN*^ brain lysates (F_(2, 20)_=126; *P*<0.0001). Rec. Tau was used as an internal control. All data represent means±s.e.m. (****P<0.0001).

As discussed above, the Tau^DN^ mutation is predicted to inhibit cleavage of Tau by caspases at D^421^ and to abolish the production of δTau. To test for this, we treated brain homogenates with recombinant Caspase-3 and analyzed samples by western blot using the anti-δTau antibody C3. This antibody is generated against the newly formed COOH-terminus of δTau and should be specific for δTau. However, C3 also cross-reacts with uncut Tau and detects full-length Tau in Caspase-3 untreated samples (Figure 2a, first two lanes). Nevertheless, Figure 2a also shows that treatment with Caspase-3 of brain homogenates isolated from WT mice, but not of brain homogenates isolated from *Tau*^*DN/DN*^ animals, produces δTau (Figure 2a).

Next, we measured the levels of brain δTau in WT and mutant mice. As the steady-state levels of endogenous δTau are low in normal brains and undetectable by western blot (Figure 2a), we used the C3 antibody in an ELISA, a method more sensitive than the western blot. As shown in Figure 2b, δTau was readily detectable in WT brain homogenate samples. A minor positive signal was also detected in *Tau*^*DN/DN*^ homogenates and recombinant human Tau, which certainly does not contain δTau. Ordinary one-way ANOVA analysis showed a significant difference among the three samples (F_(2, 19)_=133, *P*<0.0001). Tukey’s multiple comparisons test revealed a difference between WT and *Tau*^*DN/DN*^ lysates (*P*<0.0001) and WT lysates and recombinant human Tau (*P*<0.0001), but not between *Tau*^*DN/DN*^ lysates and recombinant human Tau. These data indicate that C3 cross-reacts with uncleaved full-length Tau to some degree, and suggest that the ELISA signal observed in *Tau*^*DN/DN*^ brain lysates is due to cross-reactivity with endogenous Tau^DN^. It is worth noting that this cross-reactivity was also seen in the western blot analysis shown in Figure 2a. Overall, the experiments indicate that Tau^DN^ is not cleaved by Caspase-3 at position D^421^.

### Learning and memory deficits in *Tau*^*DN/DN*^ and *Tau*^*DN/WT*^ mice

The Tau D^421^N mutation allows us to investigate the role of caspase cleavage of Tau in learning. As C57BL/6J mice are not ideal for behavioral studies, *Tau*^*DN/DN*^-C57BL/6J mice, which were obtained from the cross of founders #156 and 160, were crossed to B6129PF1/J mice (Jackson Laboratory, Cat. #100492), which are F1 hybrid mice offspring of a cross between C57BL/6J females (B6) and 129P3/J males (129P). Male and female offspring (*Tau*^*DN/WT*^ B6129PF2/J mice) mice were crossed to generate *Tau*^*DN/WT*^, *Tau*^*DN/DN*^ and WT B6129PF3/J littermates. Behavioral experiments were conducted by using male B6129PF3/J littermates as subjects and were initiated at 6 months of age, when *Tau*^*DN/WT*^, *Tau*^*DN/DN*^ and WT littermates were tested in rapid succession for: (1) anxiety-like behavior on the elevated zero maze; (2) short-term spatial recognition memory in the two-trial Y-maze; (3) general locomotor activity and anxiety-like behavior in the open field; (4) short-term memory in the novel object recognition test.

**Figure 3:**
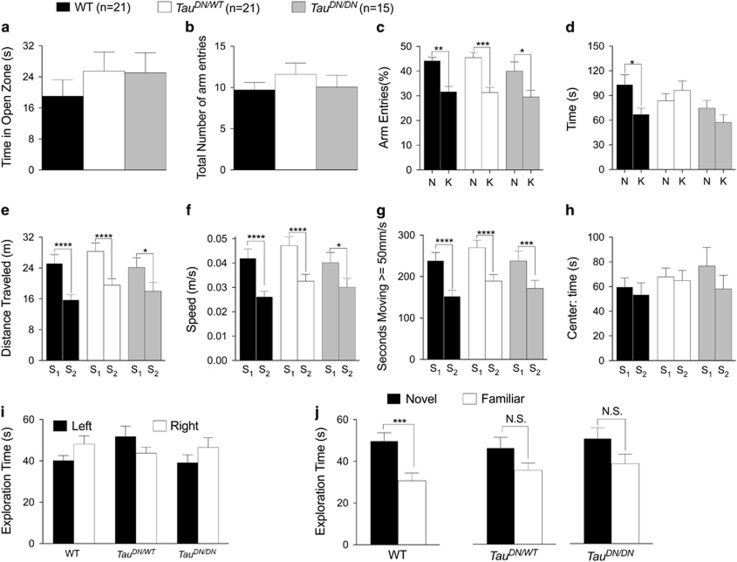
Mild short-term memory deficits in tau-mutant mice. (**a**) Elevated Zero Maze test shows no significant effect of genotype. (**b–d**) The two-trial Y-maze test showed mild deficit of short-term spatial recognition memory in *Tau*^*DN/WT*^and *Tau*^*DN/DN*^mice. (**b**) Mice of all genotypes entered the arms a similar number of times. (**c**) Percentage of entries into the novel (N) and known (K) arm. Mice of all genotypes entered the novel arm significantly more than the known arm, albeit the significance was lower for *Tau*^*DN/DN*^mice. (**d**) Time spent in the novel and known arm. Only wild-type (WT) mice and spent significantly more time in the novel arm than in the known arm. (**e–h**) Open field test shows no statistical differences among the three genotypes in total distance traveled (**e**), average speed (**f**), amount of time in which the animal ambulated at speed >50 mm s^−1^ (**g**), amount of time the animal spent in the center of the arena (**h**). In the novel object recognition task, mice of all genotypes spend similar amount of time exploring the two identical objects during the first trial (**i**). Only WT mice spent significantly more time exploring the novel object 4 h later (**j**). All data represent means±s.e.m. (*P<0.05; **P<0.01; ***P<0.001; ****P<0.0001).

One-way ANOVA showed no effect of genotype in the elevated zero maze (F_(2, 54)_=0.6145, *P*=0.5447; Figure 3a), suggesting that the Tau mutation does not cause anxiety-like behavior. In the two-trial Y-maze task, we measured the mean number of total arm entries during the 5-min test trial, which is an index for animals’ total activity levels. ANOVA found no effect of genotype, F_(2, 54)_=0.7532, *P*=0.4757 (Figure 3b). Next, we calculated the percentage of entries and the time spent into the novel (N) and known (K) arms to analyze animals’ preference for the N arm vs the K arm. As shown in Figure 3c, two-way ANOVA analysis of the percentage of entries revealed a significant main effect of arm (F_(1, 54)_=30.75, *P*<0.0001) but no differences between genotypes (F_(2, 54)_=2.728, *P*=0.0744]. Uncorrected Fisher's LSD comparisons between the arms within each genotype showed that the N arm was entered significantly more than the K arm by mice of all genotypes; however, the level of statistical significance was higher for WT (*P*<0.01) and *Tau*^*DN/WT*^ mice (*P*<0.001) than for *Tau*^*DN/DN*^ mice (*P*<0.05). In addition, two-way ANOVA analysis of the time spent into each arm showed a significant arm/genotype interaction (F_(2, 54)_=3.214, *P*=0.048]. A comparisons between the arms (uncorrected Fisher’s LSD) showed that WT mice spent significantly more time in the N arm than K arm (*P*<0.05), whereas *Tau*^*DN/WT*^ and *Tau*^*DN/DN*^ mice did not (Figure 3d). Overall, the data suggest that the D^421^N mutation may mildly compromise short-term spatial recognition memory.

In the open field test, we measured the distance traveled, the speed, the time moving ⩾50 mm s^−1^ and the time spent in the center of the arena. Two-way ANOVA indicates that mice of all genotypes were less active in the second session having familiarized with the environment (distance: F_(1, 54)_=60.60, *P*<0.0001; speed: F_(1, 54)_=61.27, *P*<0.0001; time moving ⩾50 mm s^−1^: F_(1, 54)_=85.05, *P*<0.0001; time in the center: F_(2, 54)_=0.9069, *P*<0.0001; Figures 3e–h). However, there were no statistically significant differences among the three genotypes (distance: F_(2, 54)_=1.153, *P*=0.3235; speed: F_(2, 54)_=1.160, *P*=0.3211; time moving ⩾50 mm s^−1^: F_(2, 54)_=1.232, *P*=0.2997; time in the center: F_(2, 54)_=0.5228, *P*=0.5958). These data confirm that the mutation in Tau does not cause anxiety-like behavior and suggest that it does not compromise general locomotor activity.

In the novel object recognition test, the animals were first exposed to two identical/familiar objects for 10 min, then, after an interval time of four hours, were allowed to explore the familiar object coupled to a novel one for 10 min. WT mice explored significantly more the novel object compared with the familiar one (paired *t*-test, significance at *P*=0.001). In contrast, both *Tau*^*DN/DN*^ (*P*=0.0923) and *Tau*^*DN/WT*^ mice (*P*=0.0525) exhibited no significant preference in exploring the novel object (Figure 3j), suggesting again that the D^421^N Tau mutation may mildly compromise short-term memory.

**Figure 4:**
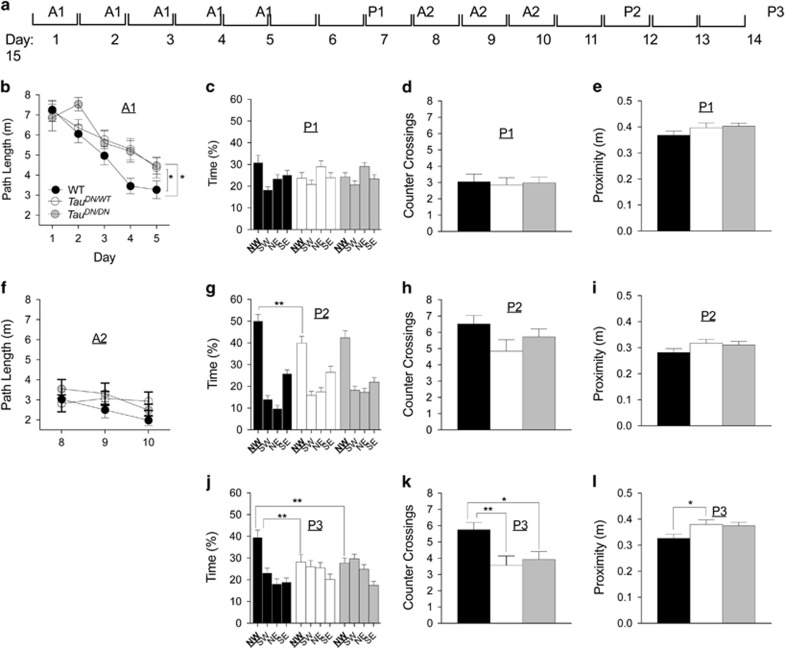
*Tau*^*DN/DN*^ and *Tau*^*DN/WT*^mice have a deficit in spatial learning and long-term memory. (**a**) Schematic illustration of the Morris water maze task. (**b**) During the first acquisition trial (A1) of spatial reference memory in the hidden platform task, a significant difference was found between WT and the two mutant mice (*Tau*^*DN/WT*^and *Tau*^*DN/DN*^), with the WT mice traveling a significantly shorter path before reaching the platform. Mice of all genotypes perform similarly during the P1 (**c–e**) and A2 (**f**) trials. (**g**) Percentage of time spent in the four quadrants in the P2 trial. *Tau*^*DN/WT*^mice spent significantly less time in the target quadrant (NW in bold) as compared with WT mice. There were no significant differences in the number of counter crossings in the target quadrant (**h**) and in the average proximity to the former platform location (**i**) among the three genotypes. (**j**) In the P3 trial, *Tau*^*DN/WT*^and *Tau*^*DN/DN*^mice spent significantly less time in the target quadrant as compared with WT mice. (**l**) *Tau*^*DN/WT*^and *Tau*^*DN/WT*^mice crossed the target quadrant significantly less than WT animals. (**h**) *Tau*^*DN/WT*^mice were on average significantly farther from the former platform location as compared with WT animals. All data represent means±s.e.m. (*P<0.05; **P<0.01).

At 10/11 months of age mice were tested for spatial reference memory in the Morris water maze task. First, we conducted a visible platform task, which showed no motor (swim speed, F_(2, 54)_=0.6671, *P*=0.5174) and no visual (path length traveled, F_(2, 54)_=1.363, *P*=0.2647) deficits in mutant mice relative to WT control (Supplementary Figure 4), attesting for the feasibility of the Morris water maze test. The task was performed as follows: (1) five-days long acquisition of the hidden platform task (referred to as A1); (2) probe trial conducted 2 days later (P1, at day 7); (3) second 3 days long acquisition trial (A2, days 8–10); (4) probe trial run 2 days later (P2, day 12); (5) last probe trial conducted after an additional 3 days (P3, day 15) (see scheme in Figure 4a).

Two-way ANOVA revealed a significant main effect for day and genotype on path length during A1 (F_(4, 270)_=21.37, *P*<0.0001; F_(2, 270)_=5.579, *P*=0.0042) (Figure 4b). Tukey’s multiple comparisons test revealed a difference with *Tau*^*DN/WT*^and *Tau*^*DN/DN*^ mice traveling a significantly larger distance than WT mice (*P*<0.05). These data indicate that the *Tau*^*DN*^ mutation partially compromises acquisition of reference memory for the platform location.

The P1 probe trial revealed a significant main effect for quadrant (percentage of time spent in the four quadrants, F_(3, 162)_=4.560, *P*=0.0043), but no significant main effect for genotype (F_(2, 54)_=0.1782, *P*=0.8372) (Figure 3c). One-way ANOVA revealed no significant effect of genotype on the number of counter crossings in the target quadrant (F_(2, 54)_=0.05494, *P*=0.9466) and on the average proximity to the original platform location (F_(2, 54)_=1.385, *P*=0.2591) (Figures 3d and e).

Two-way ANOVA revealed no significant main effect for day (F_(2, 162)_=2.221, *P*=0.1118) and genotype (F_(2, 162)_=1.862, *P*=0.1587) during the A2 task (Figure 4f). On the P2 probe trial, the analysis of the percentage of time spent in the four quadrants revealed a significant main effect for quadrant (F_(3, 162)_=78.21, *P*<0.0001), no significant main effect for genotype (F_(2, 54)_=0.3026, *P*=0.7402) but a significant quadrant × genotype interaction (F_(6, 162)_=2.935, *P*=0.0096) (Figure 4g). *Post hoc* Tukey’s multiple comparisons test showed that the *Tau*^*DN/WT*^ mice spend significantly less time in the target quadrant as compared with littermates (Figure 4g). One-way ANOVA revealed no significant effect of genotype on the number of counter crossings in the target quadrant (F_(2, 54)_=2.221, *P*=0.1183) and on the average proximity to the original platform location (F_(2, 54)_=1.919, *P*=0.1566) (Figures 4h and i). On the final P3 probe trial, two-way ANOVA of the time spent in the quadrants showed a significant main effect for quadrant (F_(3, 162)_=10.26, *P*<0.0001), no significant main effect for genotype (F_(2, 54)_=2.759, *P*=0.0723) but a significant genotype × quadrant interaction (F_(6, 162)_=2.946, *P*=0.0094). *Post hoc* Tukey’s multiple comparisons test revealed a significant difference between WT and *Tau*^*DN/DN*^ mice, and between WT and *Tau*^*DN/WT*^ mice with the homo- and heterozygous mutants spending an amount of time in the target quadrant comparable with the adjacent quadrants (Figure 4j). One-way ANOVA of the counter crossings revealed a significant main effect of the genotype (F_(2, 54)_=5.720, *P*=0.0056). A *post hoc* Tukey’s multiple comparisons test showed that WT mice cross the target platform area significantly more than *Tau*^*DN/WT*^ (*P*<0.01) and *Tau*^*DN/DN*^ (*P*<0.05) (Figure 4k). Also the average proximity to the original platform location showed a genotype effect (F_(2, 54)_=3.594, *P*=0.0343), with the *Tau*^*DN/WT*^ mice being more distant from the platform as compared to WT animals (Tukey’s multiple comparisons test, *P*<0.05) (Figure 4l).

### Synaptic plasticity deficit in *Tau*^*DN/DN*^ mice

**Figure 5:**
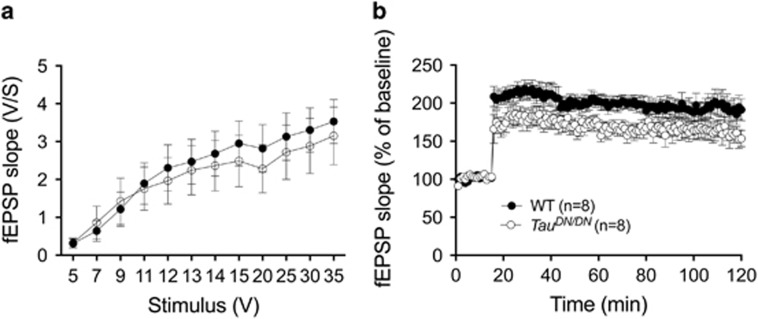
Long-term potentiation (LTP) is impaired in ~18 months-old *Tau*^*DN/DN*^mice. (**a**) Normal basal synaptic transmission in *Tau*^*DN/DN*^ animals. Summary graph of field input–output relationship for different stimulation intensities (5–35 V). Two-way ANOVA showed no difference between the two genotypes. (**b**) LTP impairment in *Tau*^*DN/DN*^animals (F_(1, 14)_=5.390, *P*=0.0359). CA1 field-excitatory postsynaptic potentials (fEPSPs) were recorded in the Schaffer Collateral pathway in the hippocampus. A 20-min baseline was recorded every minute at an intensity that evoked a response of ∼35% of the maximum evoked response. LTP was induced using a theta-burst stimulation (four pulses at 100 Hz, with the bursts repeated at 5 Hz and each tetanus including 3 ten-burst trains separated by 15 s). Responses were measured as fEPSP slopes expressed as percentage of baseline and were recorded for 2 h after tetanization.

Next, we have investigated the role of caspase cleavage of Tau in synaptic plasticity. Experimental evidence supports a role for synaptic dysfunction underlying subtle memory changes in AD. The learning/memory deficits of *Tau*^*DN/DN*^ mice prompted us to investigate synaptic transmission and plasticity using the Schaffer collateral pathway in hippocampal slices from WT and *Tau*^*DN/DN*^ mice. These tests were performed after the behavioral studies when the mice where ~18 months of age. Basal synaptic transmission was determined by measuring the slope of the field-excitatory post synaptic potential (fEPSP). We found no difference in basal synaptic transmission between WT and *Tau*^*DN/DN*^ mice (Figure 5a). However, LTP, a long-lasting form of synaptic plasticity that is associated with learning and memory, was reduced in *Tau*^*DN/DN*^ mice as compared with WT littermate control animals (F_(1,14)_=5.390, *P*=0.0359) (Figure 5b). Thus, the *Tau*^*DN/DN*^ mutation compromises LTP in older mice.

### Increased total and phosphorylated insoluble Tau in the forebrain of *Tau*^*DN/DN*^ mice

Given the surprising result shown in Figure 1d and to test the effect of the mutation on Tau phosphorylation/pathology, in experiments parallel to the synaptic plasticity study, we analyzed Tau solubility and phosphorylation by both immunohistochemistry and ELISA.

**Figure 6:**
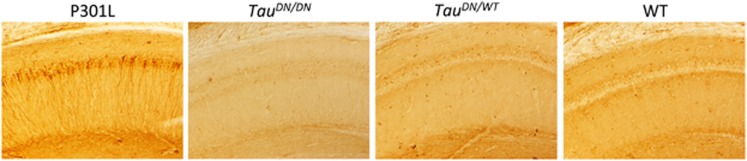
Absence of obvious Tau pathology in ~18 months-old *Tau*^*DN/DN*^and *Tau*^*DN/WT*^ mice. The CA1 hippocampal region of P301L mice (7 months-old), *Tau*^*DN/DN*^, *Tau*^*DN/WT*^ and wild-type (WT) littermates are shown in these panels. PHF1 (pSer^396-404^) immune-reactivity shows evident tau pathology in the hippocampal pyramidal cells of the P301L animals but not in the *Tau*^*DN/DN*^, *Tau*^*DN/WT*^ and WT mice.

Immunohistochemistry using the PHF1 (pSer^396–404^) did not show NFT-like lesions in neither *Tau*^*DN/WT*^nor *Tau*^*DN/DN*^ mice. In Figure 6, the CA1 hippocampal region, which is involved in memory formation and is where the Schaffer collateral pathway LTP was recorded, is shown to illustrate this point. The evidence that NFT-like lesions are detected in the pyramidal CA1 neurons of P301L transgenic animal, which express human Tau containing the most common FTDP-17 mutation (P301L) and develop NFTs,^45^ shows that the experiment was performed correctly (Figure 6).

**Figure 7:**
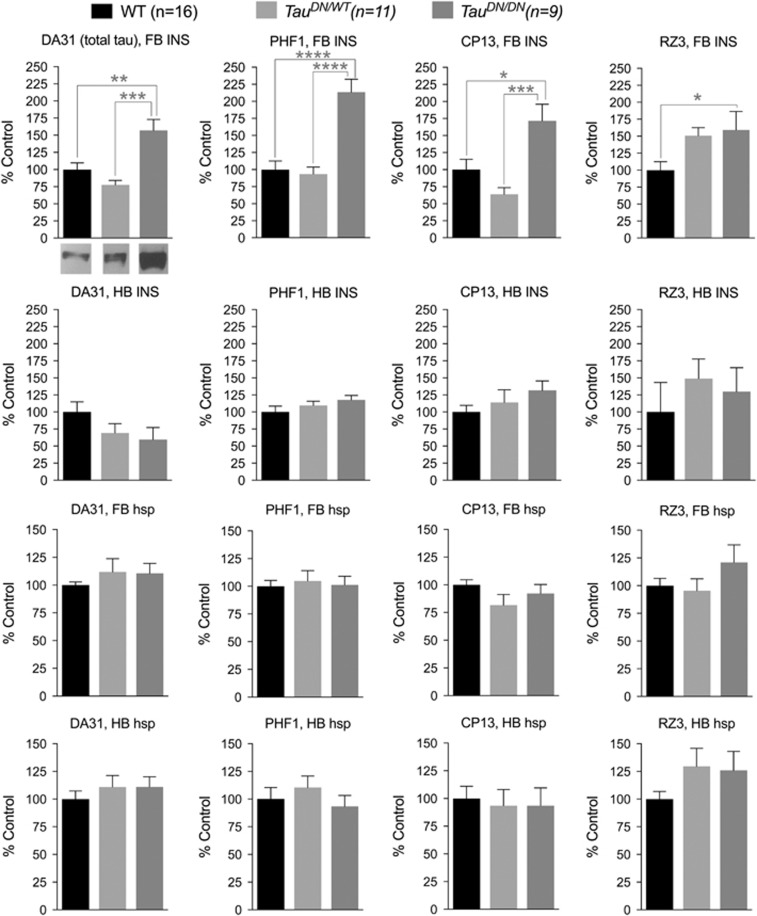
Eighteen-month old *Tau*^*DN/DN*^ mice exhibit increased insoluble Tau levels and phosphorylation in the forebrain. Total and phosphorylated levels of insoluble Tau were significantly increased in the forebrain of *Tau*^*DN/DN*^ mice compared with WT and *Tau*^*DN/WT*^ mice. Data are expressed as % of the signal seen in WT mice. FB, forebrain; HB, hindbrain: INS, insoluble; hsp, heat stable preparation (total soluble tau). A representative anti-total Tau western blot of the FB INS fractions of each genotype is shown below the graph that is located at the top-left corner of the figure. All data represent means±s.e.m. (*P<0.05; **P<0.01; ***P<0.001; ****P<0.0001).

Biochemical analysis on soluble and insoluble fractions did not show any difference in total or phosphorylated Tau, in the hindbrain area, among WT, *Tau*^*DN/WT*^and *Tau*^*DN/DN*^ mice. Also, no difference was detected in forebrain soluble fractions among the three genotypes. Interestingly enough, total and phosphorylated Tau were significantly increased in the forebrain of *Tau*^*DN/DN*^ mice when analyzing the insoluble preparation: analysis of the phosphorylation panel showed an increased overall reactivity for Ser^396–404^ (PHF1), Ser^202^ (CP13) and Thr^231^ (RZ3), with Ser^396-404^ being the site with the strongest effect (Figure 7). These data suggest that the increased levels of total brain Tau present in young *Tau*^*DN/DN*^ mice (Figure 1d) may be caused by increased insolubility of Tau in the forebrain. Future studies will have to directly address this possibility. Nevertheless, the fact that insoluble Tau levels are elevated only in the homozygous but not heterozygous KI mice (with the exception of the RZ3 antibody), whereas cognitive deficits in novel object recognition and water maze were detected in both would appear to suggest that these behavioral deficits are not dependent on accumulation of insoluble phosphorylated Tau.

## Discussion

Here we present evidence that substitution of Aspartate^421^ of mouse Tau with an Asparagine has the following effects: (1) Increases levels of total brain Tau protein (Figure 1d); (2) abolishes cleavage of Tau by caspases at D^421^, thereby preventing the formation of δTau (Figure 2); (3) induces memory deficits (Figures 3 and 4); (4) causes deficits in synaptic plasticity (Figure 5); (5) leads to an increase in total insoluble Tau and insoluble phosphorylated Tau in the forebrain (Figure 7).

On the basis of the common knowledge that δTau starts Tau pathology, our results are unexpected. Several possibilities could explain our results without negating the pathogenicity of δTau. First, our studies are related to endogenous mouse Tau and may not be entirely applicable to human Tau, as discussed in the introduction. Second, the D^421^N mutation may *per se* change the properties of Tau—leading to behavioral, synaptic and pathological changes—via a mechanism that is independent of the inhibitory effect on δTau production.

The straightforward interpretation of our data is however not consistent with the hypothesis that δTau is toxic. In contrast, the results suggest that caspase cleavage of Tau may protect from accumulation of insoluble hyper-phosphorylated Tau. These observations imply that preventing δTau formation could cause behavioral, synaptic and biochemical alterations approximating those observed in some animal models of tauopathy.^45, 46, 47, 48^ Therefore, cleavage of Tau at D^421^could represent a mechanism aimed to prevent hyper-phosphorylation and precipitation of Tau.

Finally, it is possible that both a reduction, which is the case in our mouse model—or an excess, which occurs in AD and other tauopathies—in δTau lead to synaptic dysfunction, memory impairments and Tau pathology. In this regard, it will be worth testing whether the *Tau*^*DN*^ allele in heterozygosis prevents memory and synaptic deficits in KI mouse models of human dementias characterized by increased APP processing and tauopathy.^41, 49, 50, 51, 52, 53, 54, 55, 56, 57^

Four things are worth noting concerning the alterations of Tau. First, we see an increase in total brain Tau levels in *Tau*^*DN/DN*^ mice, even at young age. This increase seems to be restricted to the insoluble fraction of the forebrain, at least in old mice. Second, Ser^396/404^ phosphorylation, which is a modification of Tau typical of late Alzheimer’s brains, is particularly elevated in the forebrain of *Tau*^*DN/DN*^ mice, compared with the other genotypes, whereas the earlier phosphorylation show a lesser increase. It is possible that analysis of younger mice would show a different pattern of Tau phosphorylation, with less PHF1 and more CP13 or RZ3 signal. We have so far focused on older mice as they were the more likely to show significant differences. Third, insoluble phospho-Tau is increased in the forebrain but not the hindbrain of *Tau*^*DN/DN*^ mice. Fourth, memory deficits in novel object recognition and Morris water maze tasks are evident in both *Tau*^*DN/WT*^ and *Tau*^*DN/DN*^ mice, albeit only *Tau*^*DN/DN*^ animals show significant alterations in forebrain insoluble Tau. This lack of correlation between biochemical and behavioral changes may suggest that the biochemical Tau alterations leading to memory deficits in *Tau*^*DN/DN*^ and *Tau*^*DN/WT*^ mice are structurally and functionally unrelated to Tau forms leading to Tau pathology in humans. Alternatively, it is possible that other Tau species, such as Tau oligomers, mediate neurotoxicity.^58, 59, 60, 61^

In this study, we have analyzed the consequence of caspase cleavage of Tau at D^421^; however, other minor caspase cleavage sites of Tau have been reported but their significance has been scantly investigated, with the exception of caspase-2 cleavage of tau at Asp314, which produces a toxic Deltatau314 peptide.^62^

Given the current on Tau as a target for therapeutic intervention, it is important to better understand the mechanisms of Tau toxicity. The data reported here seem to contradict the common knowledge that δTau is a toxic Tau species and therefore a potential therapeutic target. This possibility warrants further investigation, including an in depth analysis of the time-course and the presence or absence of sex differences in the behavioral, synaptic plasticity and biochemical deficits observed in *Tau*^*DN*^ mice.
