# Correction: Abolishing Tau cleavage by caspases at Aspartate^421^ causes memory/synaptic plasticity deficits and pre-pathological Tau alterations

**DOI:** 10.1038/s41398-018-0188-5

**Published:** 2018-08-31

**Authors:** F. Biundo, C. d’Abramo, M. D. Tambini, H. Zhang, D. Del Prete, F. Vitale, L. Giliberto, O. Arancio, L. D’Adamio

**Affiliations:** 1 Department of Microbiology and Immunology, Albert Einstein College of Medicine, Bronx, NY USA; 2 Litwin-Zucker Center for Research in Alzheimer’s Disease, Feinstein Institute for Medical Research, Northwell Health, Manhasset, NY USA; 3 Department of Pathology and Cell Biology and Taub Institute for Research on Alzheimer’s Disease and the Aging Brain, Columbia University, New York, NY USA

Correction to: *Translational Psychiatry*; 10.1038/tp.2017.165; published online 8 August 2017.

This article was originally published under Nature Research’s License to Publish, but has now been made available under a CC BY 4.0 license. The PDF and HTML versions of the article have been modified accordingly.

