# Supplementary material for: Abolishing Tau cleavage by caspases at Aspartate421 causes memory/synaptic plasticity deficits and pre-pathological Tau alterations
Source: Transl Psychiatry. 2017 Aug 8;7(8):e1198–. doi: 10.1038/tp.2017.165 (PMC5611732; doi:10.1038/tp.2017.165)
Supplement: Supplementary Information [file tp2017165x1.docx]

###### Supplementary Figure 1. Strategy used to generate *Tau^DN^* KI mice. a, Targeting Vector Schematic. b, Schematic representation of the strategy used for the generation of the point mutation.

**Supplementary Figure 2**. **Confirmation of the introduction of the point mutation in ES cells. a**, Schematic of the strategy used for PCR genotyping and confirmation of the point mutation. **b**, Results of PCR genotyping using primers F3/A2. An “x” denotes expanded clones. DNA from an individual clone (before reconfirmation) was used as a positive control and denoted as a (+). No DNA was used as a negative control, and denoted by a (--). Wild Type DNA was used as a negative control, and denoted by a wt. **c**, Confirmation of the point mutation was performed by PCR using the SQ1 and N2 primers. This reaction produces a product 0.94 Kb in size.

**Supplementary Figure 3**. **Generation of *Tau^DN^* KI mice**. **a**, Schematic of the strategy used for analysis of deletion on the Neo cassette, presence of the point mutation, presence of the FLP transgene and confirmation of the SA integration. **b**, Results of PCR using primers NDEL1/NDEL2. PCR of the Neo deleted allele yields a band of 1.06Kb, PCR of the WT allele yields a band of 872bp. **c**, Results of PCR using primers SQ1/F7. SQ1 is located on the LA, 5’ of the point mutation. F7 is located inside the Neo cassette. The amplified size for SQ1/F7 is 732 bp. Sequencing was performed on purified PCR DNA to confirm presence of the point mutation using the SQ1 primer. **d**, Results of PCR using primers FLP1/FLP2. The amplified product for primer set FLP1 and FLP2 is 725bp. **e**, Results of PCR using primers F3/A2. F3 is located inside the Neo cassette and A2 is located downstream of the SA, outside the region used to create the targeting construct. F3/A2 amplifies a fragment of 2.28 Kb in length.

**Supplementary Figure 4**. **No locomotor and visual deficits in KI mice**. Results for the visible platform task. Data are expressed as means ± S.E.M. There are no significant differences among the genotypes in speed (**a**) or in path length traveled (**b**).
