# Supplementary figures and images for: Abolishing Tau cleavage by caspases at Aspartate421 causes memory/synaptic plasticity deficits and pre-pathological Tau alterations
Source: Transl Psychiatry. 2017 Aug 8;7(8):e1198–. doi: 10.1038/tp.2017.165 (PMC5611732; doi:10.1038/tp.2017.165)

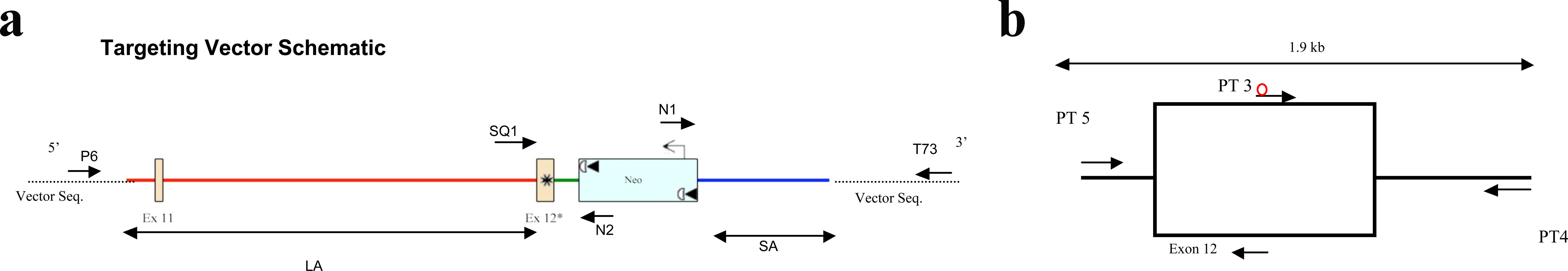

Supplement: Supplementary Figure 1 [file tp2017165x2.tif]

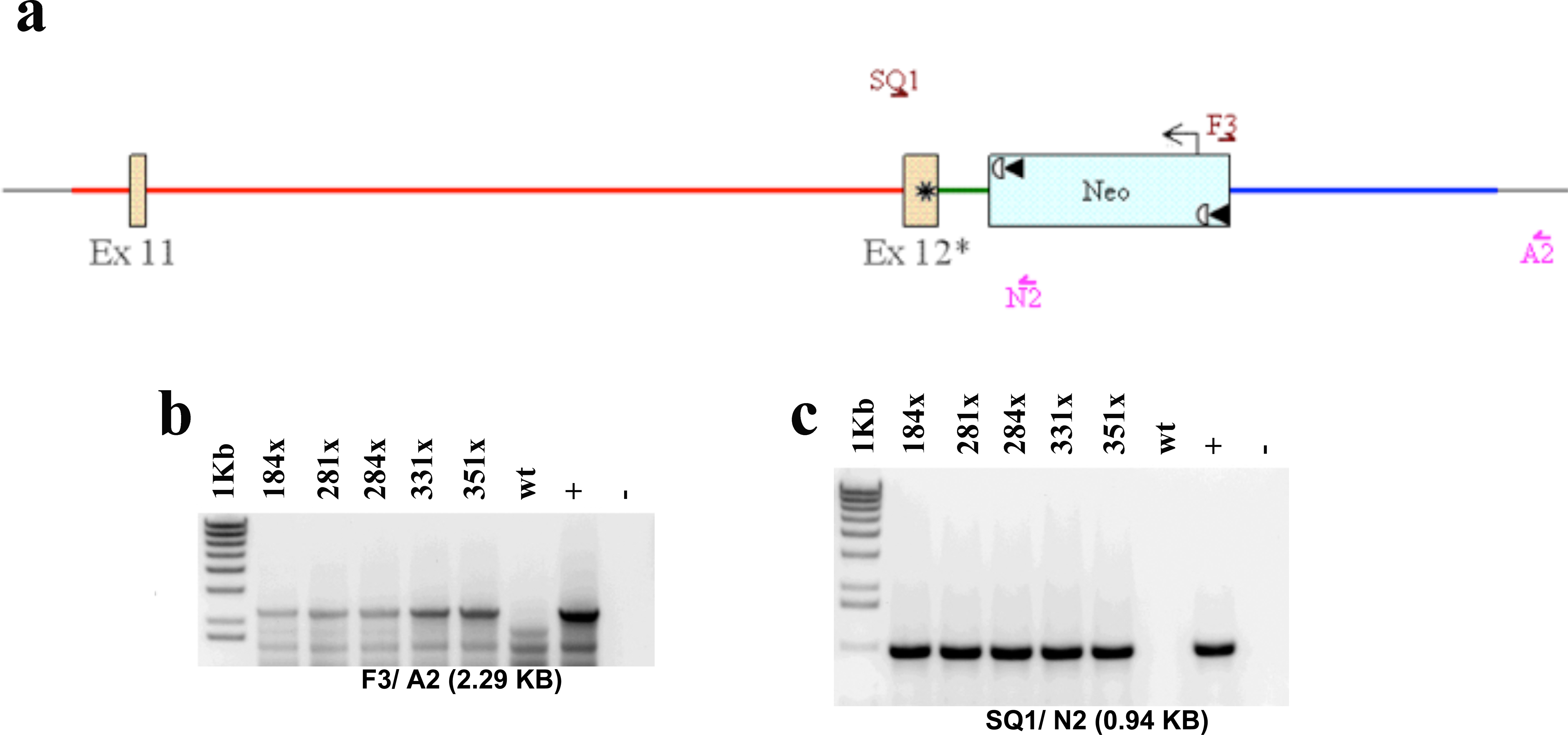

Supplement: Supplementary Figure 2 [file tp2017165x3.tif]

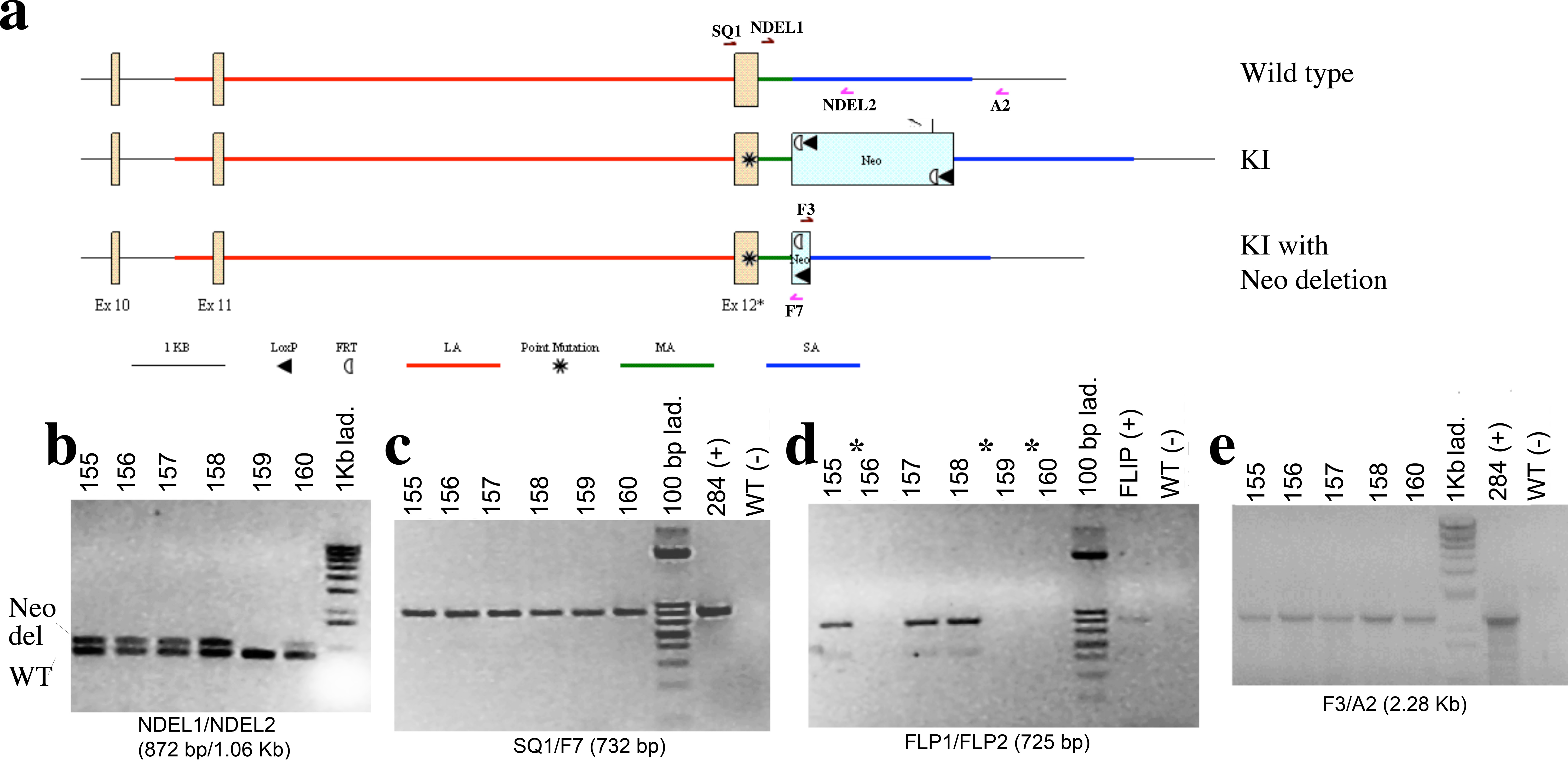

Supplement: Supplementary Figure 3 [file tp2017165x4.tif]
